# Associations between conflicting nutrition information, nutrition confusion and backlash among consumers in the UK

**DOI:** 10.1017/S1368980021000124

**Published:** 2021-04

**Authors:** Santosh Vijaykumar, Andrew McNeill, Joshua Simpson

**Affiliations:** 1 Northumbria University, Newcastle upon Tyne NE1 8ST, UK; 2 British Army, Salisbury, UK

**Keywords:** Nutrition, Media, Conflicting information, News, Confusion, Backlash, Survey

## Abstract

**Objective:**

To examine the effects of exposure to conflicting nutritional information (CNI) through different forms of media on nutrition-related confusion and backlash among consumers in the UK.

**Design:**

Cross-sectional survey administered via Qualtrics among 18–75-year-old participants in the UK. The sample was stratified by age and gender with quotas defined according to the 2011 UK census distribution.

**Setting:**

Qualtrics’ Online panel of respondents in the UK.

**Participants:**

676 participants comprising nearly an equal number of females (*n* 341) and males (*n* 335) and a majority (58·6 %) from households whose income was <£30 000.

**Results:**

Our findings showed that nearly 40 % of respondents were exposed to some or a lot of CNI. We found that while exposure to CNI from TV and online news increased nutrition confusion, CNI from health professionals increased backlash. Exposure to CNI from social media and health websites was associated with reduced backlash. We also found that nutrition confusion and backlash were negatively associated with exercise behaviour and fruit and vegetable consumption, respectively.

**Conclusions:**

Our study supports the theoretical pathways that explain the influence of CNI exposure on nutrition-related cognitive and behavioural outcomes. Additionally, different types of online information sources are associated with these outcomes to varying degrees. In the context of obesity and diabetes rates in the UK, our findings call for (a) further experimental research into the effects of CNI on consumers’ diet-related cognitions and behaviours and (b) multi-stakeholder, interdisciplinary approaches to address this problem.

Two research papers by nutrition researchers in 2019 grabbed the headlines setting off a cascade of chatter on social media platforms. The first discovered that those on plant-based diets – hitherto considered healthy – were at increased risk of stroke^(1)^. The second concluded that red meat, widely considered a risk factor for many cancers and heart disease, posed minimal danger to our health^(2)^. In addition to affecting trust and credibility in the messages and their sources among consumers^(3–5)^, conflicting nutritional information (CNI) attracts media coverage^(6,7)^ and prompts several questions of relevance to the general public who typically follow the release of findings of this nature^(8–10)^. For instance, should we now be more concerned about consuming plant-based diets and less concerned about consuming processed red meat? Should we heed the findings of nutrition scientists if they are poised to continue contradicting each other? And should we change our dietary behaviours based on the information we receive from news and social media given how inconsistent it is? The aim of this paper is to examine the extent to which exposure to CNI from various information sources shapes nutrition-related cognitive responses and behaviours among consumers in the UK.

## Understanding conflicting nutritional information

Consumer’s exposure to nutrition information can be understood to be enshrined within four overlapping spheres where such information is produced and spreads through society. In the socio-cultural sphere, consumers are engaged in a constant process of negotiating meaning, norms and beliefs related to food and dietary practices that are passed on to them through communication with their families and external social networks which include everyone from friends and relatives to personal dietitians and physicians^(11–14)^. The second sphere pertains to the food industry which produces and disseminates nutrition information through the use of nutrition labels on various food products and the strategic placement of different types of claims (health or affective) on product labels^(15,16)^. Nutrition labels and health claims have invited extensive scholarly attention and debate. This is because various studies have demonstrated consumers’ inability to easily interpret the information in these labels^(17,18)^. Additionally, many claims have proven to be unregulated or scientifically unproven, making consumers vulnerable targets to the power of creative marketing strategies^(19)^. The third sphere pertains to the digital or online world which is populated by independent nutrition websites (both generic and niche), food bloggers, food activists, social media influencers, food celebrities and governmental food agencies each of whom produces information that is based on one or a combination of personal knowledge, market insights or scientific research^(20,21)^. The last sphere, and the one of interest to this paper, is nutrition information produced by the global community of nutrition researchers. In its typical life cycle, the evidence of the health-related effects of certain foods is generated by nutrition scientists and is disseminated to scholarly audiences through research papers in academic journals and to the general public via a press release to the news media. The media’s framing of this news is driven by the agendas of media institutions and various gatekeepers such as reporters and editors and, as such, eventually shape public opinion^(22–25)^. In the social media age, this coverage diffuses rapidly through online social networks triggering an avalanche of public response and ephemeral debates along the way as seen in the cases of plant-based foods and red meat^(26)^. It is in this context that the problem of CNI takes root as nutrition research produces findings that, at many times, are at odds with each other.

CNI can be defined as information offering both positive and negative support on the nutritional effects of consuming a certain foods^(27,28)^. Consumers may thus receive information on both the risks and the benefits within the same or different messages often leading to confusion about certain commonly consumed foods such as eggs, red meat or wine, their nutritional value and ultimately whether they are beneficial or harmful to health. Although widespread, the problem of CNI is understudied and is increasingly becoming one of the public concerns in the European region. For instance, a study of consumers from eight European countries found participants perceiving risk messages about red meat as less credible when it was followed by a message about the benefits of red meat^(4)^. In the UK, conflicting signals from recommendations and guidelines, advice from health professionals and their own reading of their infants’ signs have left new mothers grappling with the decision about when to administer solid foods to their infant^(29)^. Qualitative inquiries have revealed how consumers might become vulnerable to the appeal of commercially driven nutrition information such as advertisements when confronted by conflicting or inconsistent information^(30)^. More recently, a 2018 survey of 500 adults reported that 43 % of respondents had difficulties in finding reliable information on healthy diets and that nutrition information from the media (76 %) and experts (61 %) caused most confusion^(31)^. A measure of the concern that CNI poses is the launch of television programmes such as BBC’s ‘Food: Truth or Scare’ aimed at helping its audience sift through CNI^(32)^ and other online resources developed to enable consumers to decipher health-related news in the media initiated by the British Nutrition Foundation^(33)^ and the NHS^(34)^. The public concern about CNI, as evidenced by these examples, is problematic when the current context is considered: the UK faces an obesity crisis and therefore requires the majority of the population to make critical dietary choices; and social media, the most preferred source of nutrition information, is now rife with health-related misinformation^(31,35,36)^.

## Problem definition and theoretical framework

Three inter-related and complex phenomena underpin the CNI problem. The evolutionary and incremental nature of the scientific process renders the discovery of conflicting or contradictory evidence integral to the nutrition research enterprise^(28)^. Different forms of media (e.g. print, radio, the Internet) exert different levels of influence on nutrition-related attitudes and behaviours^(37,38)^. And lastly, the sheer multiplicity of and interlinkages between health information sources within the Internet – online news, specialised medical websites and social media conversations – mean that consumers are now grappling with an ‘infodemic’, a term that denotes excessive amounts of information including conflicting information and misinformation^(39)^. Because consumers’ trust could vary from one source to the other based on perceptions of credibility, their ability to act upon such information is compromised^(40–42)^.

As a first step, it is important to develop an understanding of the breadth of the CNI problem and the burden it imposes on individual-level perceptions and decisions surrounding food, diet and nutrition. For instance, we have yet to understand the prevalence of exposure to CNI among UK consumers and the extent to which such exposure varies by media type. The pathways between CNI exposure and health attitudes and behaviours assume salience in health emergency situations like COVID-19 where informational lacunae combine with evolving scientific findings to create informational uncertainties for individuals eventually resulting in distrust of policymakers^(43,44)^. In England, COVID-19 has been shown to disproportionately affect those with diet-related conditions like type 2 diabetes^(45)^. It is important to create an evidence base that can inform the practice of nutrition communicators looking to alleviate decisional conflict and ambiguity among such vulnerable groups.

In order to address these gaps in research, our study builds on the work of Lee *et al.* (2018) who investigated the cognitive and behavioural effects of CNI through a three-wave panel study in the USA. Specifically, the authors investigated the variable influences of different types of media sources on (a) nutrition confusion – defined as perceived ambiguity about nutrition recommendations and research and (b) nutrition backlash – defined as negative beliefs about nutrition recommendations and research. They further studied the extent to which nutrition confusion and backlash affected fruit and vegetable consumption and exercise-related behaviours. The authors built a model of effects which demonstrated that exposure to CNI via television and print media significantly influenced nutrition confusion, nutrition confusion significantly influenced nutrition backlash and nutrition backlash was related to decreased fruit and vegetable consumption. We sought to adapt this study by testing the aforementioned pathways in the UK context where discussion about CNI has seldom ventured beyond descriptive studies, media commentaries and discussions on online forums.

There are however two key differences in our study. One, we employed a cross-sectional design as opposed to using a multiple wave design as was done in the original study. This decision was purely driven by available resources. Two, the original authors aggregated CNI-related perceptions related to online news, social media and medical/health websites and provided a singular score for CNI from the ‘Internet’. However, we suggest that these three sources have different functions: online news presents health information for a generic audience, health/medical websites aim to provide accurate and unbiased information on all aspects of the topic for an audience looking for information on specific health topics and social media uses the underlying infrastructure of social networks to diffuse information that could be aggregated from different kinds of sources including online news and medical websites^(46,47)^. Hence, in our study, we will assess the results of two models, one which combines the three sources (similar to the original study) and the other which examines their effects individually.

## Study hypotheses

The hypotheses of the original study are listed below. Our own additional hypothesis is in italics.

H1. There will be a positive relationship between exposure to CNI sources and confusion.

*H1a. When CNI received via the Internet is segregated by source, there will be different effects on confusion for each source.*

H2. There will be a positive relationship between confusion and backlash.

H3. There will be an indirect effect of CNI on backlash via confusion.

H4. Confusion will be linked to lower fruit and vegetable consumption.

H5. Confusion will be linked to lower exercise frequency.

H6. Backlash will be linked to lower fruit and vegetable consumption.

H7. Backlash will be linked to reduced exercise frequency.

H8. There will be an indirect effect of confusion on fruit and vegetable consumption via backlash.

H9. There will be an indirect effect of confusion on exercise frequency via backlash.

## Methodology

We conducted a cross-sectional online panel survey administered via Qualtrics among 18–75-year-old participants in the UK. The sample was stratified by age and gender with quotas defined according to the 2011 census distribution for these demographic variables. The survey was first piloted (*n* 51) to identify discrepancies which were later rectified in the final questionnaire (*n* 676). The age and gender distribution of the respondent pool was representative of the UK population with participants predominantly employed and white. A full breakdown of the participant sample is given in Table 1. All data and analyses from the study are available to access at https://osf.io/zpa5b/.

**Table 1:** Socio-demographic profile and awareness of expert nutrition information sources of survey respondents (*n* 676)

Variable	Categories	Frequency	(Unweighted) Percentage
Age	18–24 years	111	16·4
	25–34 years	103	15·2
	35–44 years	119	17·6
	45–54 years	137	20·3
	55–64 years	97	14·4
	65–75 years	109	16·1
Gender	Male	335	49·6
	Female	341	50·4
Occupational status	Employed	411	60·8
	Unemployed	70	10·4
	Retired	109	16·1
	Others (Student/Self-employed/Carer/Disabled)	86	12·7
Household income	Up to £10 000	79	11·7
	£10 000–£14 999	75	11·1
	£15 000–£19 999	94	13·9
	£20 000–£29 999	148	21·9
	£30 000–£39 999	125	18·5
	£40 000 and above	155	22·9
Ethnicity	White	596	88·2
	Non-white	80	11·8
Behind the Headlines	Heard of Visited website	95 58	14·1 8·6
The Eatwell Guide	Heard of Visited website	105 61	15·5 9·0

### Measures (variables of interest)

Main outcome variables were measured as follows:

- Exposure to CNI: Participants rated the extent of CNI they received from various sources over the past 12 months on a four-point scale (‘not at all’, ‘a little’, ‘some’ and ‘a lot’). The sources specified were ‘online news’, ‘social media’, ‘medical or health websites’, ‘TV’, ‘Newspapers or magazines’, ‘Family, friends or co-workers’, ‘Doctors or other healthcare professional’ and ‘Other source’. Consistent with Lee *et al*. (2018), online news, social media and medical or health websites were combined into an average score for ‘Internet’ (*α* = 0·78).
- Nutrition Confusion: As in Lee *et al*. (2018), three items measured nutrition confusion (e.g. ‘It is not always clear to me what foods are best for me to eat’) on a four-point scale (‘strongly disagree’, ‘disagree’, ‘agree’, ‘strongly agree’). The scale had good reliability (*α* = 0·79). We opted for a four-point scale based on data from a pilot study (*n* 51) which suggested that large numbers of participants were selecting the central point on these scales (neither agree nor disagree; 23·5, 31·4 and 39·2 % for each item, respectively). Changing to a four-point scale did not affect reliability (*α* = 0·71 for the pilot). Distribution of scores from the five-point to the four-point scale did not change for the first two items (positively skewed towards ‘agree’; ‘It is not always clear to me what foods are best for me to eat’ and ‘I often find food nutrition recommendations to be confusing’) and changed from an even distribution to a positively skewed distribution for the third item (‘I find food nutrition research studies hard to follow’).
- Nutrition Backlash: Five items measured nutrition backlash (e.g. ‘I am tired of hearing about what foods I should or should not eat’) on a four-point scale (‘strongly disagree’, ‘disagree’, ‘agree’, ‘strongly agree’). One item from the scale used by Lee *et al.* (2018) was accidentally dropped from the survey (‘The evidence about healthy food choices is growing’). The scale had poor reliability (α = 0·58). Closer inspection of the items suggested that the final two items were more about attitudes to research than about reactions to recommendations (‘Scientific research provides good guidance about the best foods to eat,’ and ‘I pay attention to new research on food and nutrition’). This was borne out by an exploratory factor analysis that suggested one factor based on eigenvalues >1 (factor loadings shown in Table 2). On this basis, the backlash scale was measured by the three items that loaded onto Factor 1 and these had acceptable reliability (*α* = 0·70).
  **Table 2:** Means analysis of information sources of conflicting nutritional information (CNI), and health-related cognitions in decreasing order of mean scores

	Valid	Mean	sd
Sources of CNI (1–4)*:			
TV	674	2·48	1·04
Online news	676	2·40	1·00
Social media	676	2·30	1·15
Internet	676	2·24	0·89
Family, friends and co-workers	673	2·24	1·04
Newspapers or magazines	673	2·13	1·08
Medical health websites	674	2·02	1·04
Doctors and healthcare professionals	675	1·89	1·01
Other sources	667	1·68	0·95
Health status (1–5)	676	3·28	1·10
Fruit and vegetable daily (1–5) consumption	676	3·24	1·33
Exercise (weekly) (1–5)	676	2·45	1·21
Health consciousness (1–4)	676	2·97	0·52
Confusion (1–4)	676	2·89	0·63
Backlash (1–4)	676	2·58	0·46

* Numbers in parentheses indicate the lower and higher end points of the scale.
- Healthy Behaviours: One item measured fruit and vegetable consumption (‘In the past week, on average, how many servings of fruit, vegetables did you eat or drink per day? (This includes table juice and fresh, frozen or canned fruits/vegetables).’). This was measured on a five-point scale ranging from ‘1 or less’ to ‘5 or more’. One item measured exercise (‘During an average week, how often do you exercise?’) on a five-point scale from ‘Never’, ‘1–2 times’, ‘3–4 times’, ‘4–5 times’ to ‘6 or more times’.

### Measures (control variables)

Analysis of the theoretical model controlled for three variables per Lee *et al*. (2018). Household income was measured on a six-point scale (up to 10K, 10–14·9K, 15–19·9K, 20–29·9K, 30–39·9K, 40K+). Health status was measured on a five-point scale (Poor–Excellent). Health consciousness was measured with six items (e.g. ‘I reﬂect about my health a lot’; *α* = 0·84) on a four-point scale (‘strongly disagree’, ‘disagree’, ‘agree’, ‘strongly agree’).

### Analyses

Simple means analysis was used to measure the prevalence of CNI exposure, health status, level of health consciousness and trust in traditional and online media (Table 3). Following correlational analysis of key outcome variables, we then used the ‘*lavaan*’ package with the JASP front-end to conduct confirmatory factor analysis (see online supplementary material, Supplemental Table 1) and structural equation modelling (SEM). Consistent with Lee *et al*. (2018), we modelled confusion and backlash as latent variables with confirmatory factor analysis demonstrating their good fit with the data [(*χ*^2^(8) = 10·62, *P* = 0·22, Root Mean Square Error of Approximation (RMSEA) = 0·02, Comparative Fit Index (CFI) = 0·997, Standardised Root Mean Squared Residual (SRMR = 0·03)].

**Table 3:** Correlation matrix for main variables

	Internet	TV	Print	Lay people	Health professionals	Confusion	Backlash	Servings of fruit and vegetables
Internet		–						
TV		0·60***	–					
Print		0·54***	0·59***	–				
Lay people		0·58***	0·47***	0·47***	–			
Health professionals		0·55***	0·46***	0·41***	0·53***	–		
Confusion		0·19***	0·19***	0·16***	0·10**	0·11**	–	
Backlash	−0·03	0	−0·02	−0·11**	0·02	0·18***	–	
Servings of fruit and vegetables	0·21***	0·16***	0·12**	0·18***	0·11**	−0·03	−0·18***	–
Exercise frequency	0·13***	0·09*	0·13***	0·20***	0·14***	−0·07	−0·10**	0·21***

*P < 0·05, **P < 0·01, ***P < 0·001.

The SEM analysis comprised first running the original model (model 1) as per Lee *et al.* (2018) and then further investigate key paths in a revised model (model 2). Direct paths were drawn from each of the five information sources to nutrition confusion and nutrition backlash, from nutrition confusion to nutrition backlash, and from nutrition confusion to exercise, and nutrition backlash to fruit and vegetable consumption. Direct paths were also drawn from the control variables to mediating and dependent variables when there was a significant correlation, that is, from Household income to Fruit and Vegetable Consumption, from health status to Exercise Frequency and Fruit and Vegetable Consumption, from Health Consciousness to Exercise Frequency, Fruit and Vegetable Consumption, Confusion, and Backlash (these are not displayed in the diagrams for clarity).

## Results

### Descriptive analyses

We found that nearly 83 % of the respondents reported having encountered CNI over the past year, of which 39·9 % of participants reported being exposed to ‘some’ or ‘a lot’ of CNI (see online supplementary, Supplemental Table 2).

Table 2 provides means analyses of the levels of exposure to CNI from different information sources and various health and diet-related cognitions. We found that participants were most exposed to CNI from television, online news and social media and were least exposed to it from doctors and health professionals. Participants reported high levels of nutrition confusion and nutrition backlash to CNI, although only few of them were familiar with or used expert information sources such as NHS Behind the Headlines, BNF’s Facts Behind the Headlines and the Eatwell Guide (see Table 1) to address this problem. While we found moderate to high health status and fruit and vegetable consumption, exercise behaviour was low to moderate.

### Model testing

Consistent with our analytical approach described in the section ‘Analyses’ the correlation matrix for key variables of interest is shown in Table 3. The matrix demonstrates statistically significant linear relationships between most variables, ranging in strength from extremely weak (0·00–0·20) to moderate (0·40–0·60).

#### Model 1 (Original model)

**Fig. 1:**
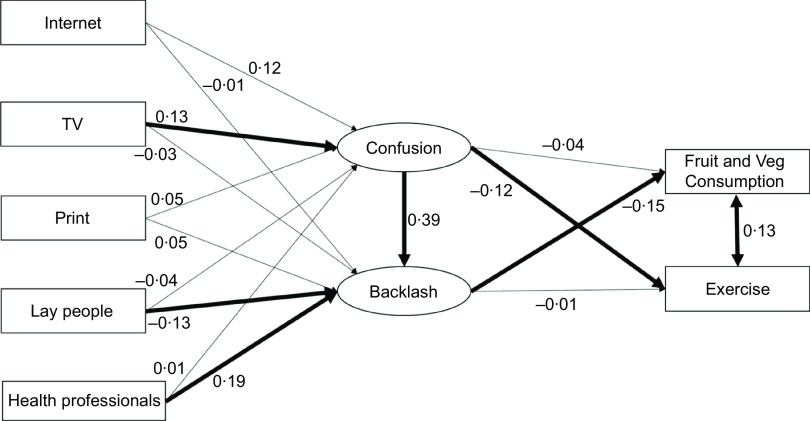
Structural equation modelling (SEM) model showing standardised estimates. Control variables and correlations between the sources of exposure are not shown for clarity. Significant paths are in bold

Direct paths were drawn from each of the five information sources to nutrition confusion and nutrition backlash, from nutrition confusion to nutrition backlash, and from nutrition confusion to exercise, and nutrition backlash to fruit and vegetable consumption. The SEM model is diagrammed in Fig. 1.

The SEM model was a good fit to the data: *χ*^2^(63) = 75·71, *P* = 0·13, RMSEA = 0·02, CFI = 0·988, SRMR = 0·02. Exposure to CNI from TV predicted confusion (*b* = 0·13, se = 0·03, *P* = 0·02; H1); confusion predicted backlash (*b* = 0·39, se = 0·05, *P* < 0·01; H2); backlash negatively predicted fruit and vegetable consumption (*b* = –0·15, se = 0·11, *P* < 0·01; H6); and there was a relationship between fruit and vegetable consumption and exercise frequency (*b* = 0·13, se = 0·04, *P* < 0·01). We found a significant pathway for the effect of lay people (*b* = –0·13, se = 0·03, *P* = 0·02) and health professionals (*b* = 0·19, se = 0·03, *P* < 0·01) on backlash (H1). Conflicting information from lay people predicted less backlash, whereas conflicting information from health professionals predicted more backlash. We also found a significant negative effect of confusion on self-reported exercise (*b* = –0·12, se = 0·10, *P* < 0·01; H5, supported here but not in previous study).

We found an indirect effect of information from TV on backlash mediated by confusion (*b* = 0·05, se = 0·01, *P* = 0·03; H3). In addition, although we did not find a significant indirect effect of CNI from lay people on fruit and vegetable consumption via backlash, we did find a significant indirect effect of health professionals on fruit and vegetable consumption mediated through backlash (*b* = –0·03, se = 0·01, *P* = 0·02; H3). We found an indirect effect of confusion on fruit and vegetable consumption via backlash (*b* = –0·06, se = 0·04, *P* < 0·01; H8) but did not find an indirect effect of confusion on exercise frequency via backlash (contrary to H9).

Consistent with the previous work, H4 (link between confusion and fruit and vegetable consumption) and H7 (link between backlash and reduced exercise) were not supported.

#### Model 2 (Revised model)

**Fig. 2:**
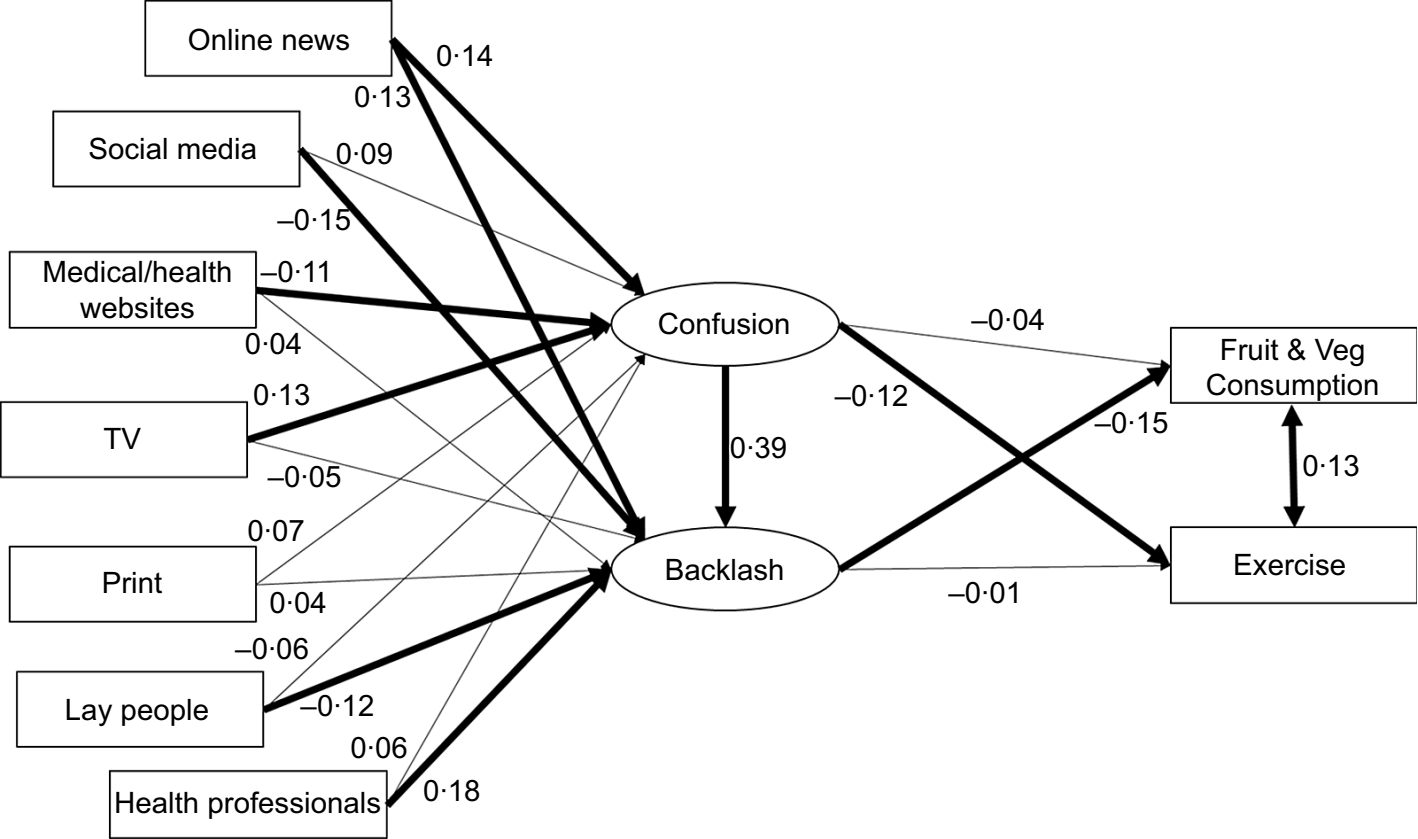
Model 2 structural equation modelling (SEM) breaking internet information into components of online news, social media and medical/health websites. The model shows standardised estimates. Control variables and correlations between the sources of exposure are not shown for clarity. Significant paths are in bold

Given that there was no effect of conflicting information received via the Internet (contrary to what we expected), we considered the possibility that different Internet sources may be perceived differently. Consequently, we ran a second SEM analysis separating out the different aspects of Internet information into information received from online news, from social media and from medical/health websites. This produced a model that was a slightly better fit (*χ*^2^(75) = 76·85, *P* = 0·42, RMSEA < 0·01, CFI = 0·998, SRMR = 0·02). This model is diagrammed in Fig. 2.

This second model shows that CNI received via the Internet does have effects on confusion and backlash. In addition to the significant paths identified in model 1, there are significant regressions on confusion from online news (*b* = 0·14, se = 0·03, *P* = 0·01) and from medical/health websites (*b* = –0·11, se = 0·03, *P* = 0·047) – consistent with H1a. Backlash is also significantly regressed by online news (*b* = 0·13, se = 0·03, *P* = 0·04) and social media (*b* = –0·15, se = 0·03, *P* = 0·01). There was also a significant indirect effect of online news on backlash via confusion (*b* = 0·06, se = 0·01, *P* = 0·02). There was no significant indirect effect of medical/health websites on backlash via confusion. Indirect relationships on both fruit and vegetable consumption and exercise were tested from significant predictors of confusion and backlash (online news, social media and medical/health websites), but no significant relationships were found. An indirect relationship between confusion on fruit and vegetable consumption via backlash was found (*b* = –0·06, se = 0·04, *P* < 0·01) but not for the relationship between confusion on exercise via backlash.

## Discussion

This paper examined the cognitive and behavioural effects of exposure to conflicting or contradictory information to food, diet and nutrition-related issues in the UK. We sought to adapt Lee *et al*.’s (2018) model which looked at the varying influence of different media sources on nutrition-related confusion and backlash, and subsequent effects on individual-level food consumption and exercise. Key findings included prevalence of exposure to CNI, confusion and backlash, and limited utilisation of online expert resources made available by the NHS and other sources. Model testing revealed not only significant pathways most of which provided support for Lee *et al*.’s (2018) findings but also new linkages that merit attention from theoretical and practical perspectives.

We found additional support for the authors’ hypotheses about a positive relationship between exposure to CNI specifically via TV, the relationship between nutrition-related confusion and backlash, the indirect effects of CNI exposure on backlash via confusion, and the negative relationships between backlash and fruit and vegetable consumption. These findings demonstrate that the influence of CNI exposure on consumer cognitions and subsequent negative effects on individual-level health behaviours that were originally found in the USA also exist in the UK.

Additionally, our analyses revealed several new significant pathways which add to the original work and provide contextual nuance – three of these pathways merit discussion. First, the original model found a negative relationship between exposure to CNI from laypeople and nutrition backlash. This finding can be explained by the potential to engage in back-and-forth exchanges with interpersonal networks which allows for real-time feedback, thereby increasing the opportunity to reconcile ambiguities^^(48,49)^^. Alternatively, interpersonal communication about CNI among lay people might culminate in deference to experts with deeper knowledge of issues, thereby reducing backlash^^(50)^^. Our data are insufficient to adjudicate between these explanations, and further exploration is needed.

Second, we found that exposure to CNI from health professionals is associated with higher backlash which is consistent with our rationale for the effect of lay persons. Since health professionals can be seen as being responsible for or identified with research, CNI from health professionals and the resultant ambiguities shape negative perceptions about research, thereby increasing backlash. Diet-related advice could be shared by health professionals during the course of a clinical interaction. However, the pervasive influence of the Internet has now meant that patients seek information from online sources both prior to, and after, the clinical interaction for seeking reassurance about advice they have just received^^(51)^^. These behaviours have been shown to result in potential dissatisfaction with the health professions and tensions in the patient–provider relationship (*ibid*).

Third, we found useful insights by trifurcating the collective ‘Internet’ variable into online news, social media and health/medical websites especially as we found that each of them discretely influenced nutrition confusion or backlash. Our approach is informed by the fact that the Internet is a diverse medium, and each of these three online sources provides distinct utilities to the user. Internet scholars suggest that consumption of health news from the websites of traditional print and broadcast news outlets is driven by generic day-to-day consumption of news^^(47)^^. An analysis of the newspaper reporting of dietary advice in the UK concluded that unsubstantiated claims made by the press ‘may contribute to public confusion regarding authoritative dietary advice’, providing heft to our finding that CNI exposure from online news shaped both nutrition-related confusion and backlash^^(52)^^. Medical/health websites, on the other hand, are mostly used by those seeking information related to specific health-related conditions^^(47)^^.

CNI from medical and health websites was associated with reduced confusion. This finding might seem counterintuitive given that the Internet is one of the most common sources of conflicting information^^(53)^^. However, a study examining online health information seeking among English women approaching menopause suggests that Internet users employ heuristics to sift through online information and assessed its credibility based on expertise and bias before placing their trust in any website^^(54)^^. In the USA, Jung and colleagues (2016) concluded that message accuracy plays a greater role in shaping perceptions of credibility among participants with high issue involvement and that those with low levels of prior knowledge tended to apprise both, the accuracy of the message and the expertise of the source. The specific heuristics and cues that UK Internet users employ in navigating the maze of conflicting online nutritional information remains an understudied area and commands further investigation. It appears from our findings that the source of CNI could have a bearing on cognitive responses. For instance, some conflicting information (e.g. that which is received from laypersons) might offer no basis for arbitrating the contradictory claims. Other conflicting information, such as that received from health and medical websites, helps to weigh up the relative merits of the conflicting claims and may thus reduce confusion.

Last, social media serves as an integrating, catalytic platform that draws information from various sources including news media and medical websites and provides a forum for real-time, albeit ephemeral debates. Our finding about exposure to CNI on social media reducing backlash needs to be considered in the context of social media being the preferred medium for nutrition information seeking in the UK^^(31)^^. In contrast to online news and medical websites, social media offers consumers the facility to communicate with their interpersonal networks as well as experts, health professionals and food and health organisations all of whom coexist in the same space. Communication between these communities of experts and consumers potentially reduces confusion, increases clarity and alleviates backlash.

While offering these suggestions for the mechanisms responsible for the relationships between different Internet sources and confusion or backlash, we acknowledge that our explanations are only tentative and merit further research to understand the mediators between receiving conflicting nutrition information via various Internet sources and its effect on confusion. Potential mediating variables to be considered include trust, self-efficacy and understanding of the scientific process which leads to the generation of findings from nutritional scientists. Additionally, recalibrating nutrition information from a five-point to four-point scale may have produced response bias in the third item, which we acknowledge as a limitation (i.e. participants may not read nutrition research studies and may genuinely neither agree nor disagree with this item).

### Implications for public health nutrition research and practice

Our paper draws attention to several issues and highlights several gaps in this area of critical concern to the public health nutrition community. The wide prevalence of exposure to CNI and associated levels of nutrition confusion and backlash commands the need to conduct a scaled up, longitudinal survey so that we can investigate the influence of geographical or socio-demographic factors on these cognitions and examine them over time. The low rates of identification and adoption of expert sources of verification suggest the need to engage audiences further and develop more interactive ways of aiding the public to sift through conflicting or contradictory nutrition information. Our addition to Lee *et al*.’s (2018) study by way of highlighting the differential and significant influences of different types of Internet sources on nutrition-related confusion and backlash opens new doors for investigation. For instance, it would be useful to use big data techniques to track the life cycle of nutrition information from the press release by scientists’ institutions to media coverage and subsequent transmission on social media. This would allow us to not only track the networks that underlie linkages between online news, medical websites and social media but also help us identify how social media influencers shape the agenda of nutrition news and contribute to its diffusion. Last, our work has continued the methodological tradition of the original work in terms of an online survey. However, it is incumbent upon the nutrition communication community to employ more scientifically robust methodologies such as experimental trials to identify heuristic cues that influence audiences’ consumption of nutrition information. These insights can be leveraged to develop ubiquitous digital interventions that can strengthen consumers’ ability to discern between news about nutrition findings that is scientifically robust or weak.

Our findings bear several implications for public health practitioners and more specifically, nutrition educators and communicators. At a policy level, it is incumbent upon governmental organisations such as the NHS to investigate the causes of limited exposure to, and use of, their online tools that seek to demystify CNI. These efforts may include examining the barriers to access or uptake of these tools and redesigning them in a manner that attracts wider engagement. There is also a need for cross-sectoral collaboration and dialogue to unpack the CNI problem by involving all stakeholders. These include scientists, public relations experts in universities who translate research findings into press releases, science journalists and non-profit civil sector organisations like the British Nutrition Foundation and the British Dietetics Association. Such collaborations may help to develop coordinated and innovative digital interventions for consumers that blend insights from psychology, design and science communication. Last, global concerns around the COVID-19 infodemic mean that the online information overload^^(55)^^ will likely expose consumers to not just CNI but also misinformation spread by both human and automated agents. In concert with our findings, these contemporary trends imply a critical need for the public health nutrition community to come up with ways to minimise confusion and backlash. As we seek to forestall the threats of technological advancements to the nutritional information environment, it will be important to do so by leveraging these new capabilities.

## Supplementary material

For supplementary material accompanying this paper visit https://doi.org/10.1017/S1368980021000124. click here to view supplementary material

## References

[1] Tong TY , Appleby PN , Bradbury KE et al. (2019) Risks of ischaemic heart disease and stroke in meat eaters, fish eaters, and vegetarians over 18 years of follow-up: results from the prospective EPIC-Oxford study. BMJ 366, l4897. 31484644 10.1136/bmj.l4897 PMC6724406

[2] Han MA , Zeraatkar D , Guyatt GH et al. (2019) Reduction of red and processed meat intake and cancer mortality and incidence: a systematic review and meta-analysis of cohort studies. Ann Intern Med 171, 711–720. 31569214 10.7326/M19-0699

[3] O’Key V & Hugh-Jones S (2010) I don’t need anybody to tell me what I should be doing’. A discursive analysis of maternal accounts of (mis) trust of healthy eating information. Appetite 54, 524–532. 20170695 10.1016/j.appet.2010.02.007

[4] Regan Á , McConnon Á , Kuttschreuter M et al. (2014) The impact of communicating conflicting risk and benefit messages: an experimental study on red meat information. Food Qual Preference 38, 107–114.

[5] Vardeman JE & Aldoory L (2008) A qualitative study of how women make meaning of contradictory media messages about the risks of eating fish. Health Commun 23, 282–291. 18569057 10.1080/10410230802056396

[6] BBC (2019) Is red meat back on the menu? https://www.bbc.co.uk/news/health-49877237 (accessed September 2019).

[7] The Guardian (2019) Being vegetarian ‘lowers heart disease risk but increases chance of stroke’. https://www.theguardian.com/society/2019/sep/04/being-vegetarian-lowers-heart-disease-risk-but-increases-chance-of-stroke (accessed September 2019).

[8] Lear S (2018) Why the heck can’t scientists agree on what is good to eat? https://blogs.bmj.com/bjsm/2018/03/26/why-the-heck-cant-scientists-agree-on-what-is-good-to-eat/ (accessed March 2018).

[9] The Washington Post (2017) Confused about what’s healthy? A new nutrition survey shows you’re not alone. https://www.washingtonpost.com/lifestyle/wellness/confused-about-whats-healthy-a-new-nutrition-survey-shows-youre-not-alone/2017/05/15/ad75f7e2-367e-11e7-b4ee-434b6d506b37_story.html (accessed May 2017).

[10] New Scientist (2019) Why everything you know about nutrition is wrong. https://www.newscientist.com/article/mg24332380-000-why-everything-you-know-about-nutrition-is-wrong/ (accessed July 2019).

[11] Bruss MB , Morris JR , Dannison LL et al. (2007) Food, culture, and family: exploring the coordinated management of meaning regarding childhood obesity. Health Comm 18, 155–175. 10.1207/s15327027hc1802_4 16083409

[12] Kaplan M , Kiernan NE & James L (2006) Intergenerational family conversations and decision making about eating healthfully. J Nutr Educ Behav 38, 298–306. 16966051 10.1016/j.jneb.2006.02.010

[13] Visser SS , Hutter I & Haisma H (2007) Building a framework for theory-based ethnographies for studying intergenerational family food practices. Appetite 97, 49–57. 10.1016/j.appet.2015.11.019 26593100

[14] Wright LT , Nancarrow C & Kwok PM (2001) Food taste preferences and cultural influences on consumption. Br Food J 103, 348–357.

[15] Colby SE , Johnson L & Scheett A (2010) Nutrition marketing on food labels. J Nutr Educ Behav 42, 92–98. 20096635 10.1016/j.jneb.2008.11.002

[16] Van Camp D , de Souza Monteiro DM & Hooker NH (2012) Stop or go? How is the UK food industry responding to front-of-pack nutrition labels? Eur Rev Agric Econ 39, 821–842.

[17] Cowburn G & Stockley L (2005) Consumer understanding and use of nutrition labelling: a systematic review. Public Health Nutr 8, 21–28. 15705241 10.1079/phn2005666

[18] Grunert KG & Wills JM (2007) A review of European research on consumer response to nutrition information on food labels. J Public Health 15, 385–399.

[19] Hoffmann D & Schwartz J (2016) Stopping deceptive health claims: the need for a private right of action under federal law. Am J Law Med 42, 53–84. 27263263 10.1177/0098858816644715

[20] Jung EH , Walsh-Childers K & Kim H-S (2016) Factors influencing the perceived credibility of diet-nutrition information web sites. Computers Hum Behav 58, 37–47.

[21] Rutsaert P , Regan Á , Pieniak Z et al. (2013) The use of social media in food risk and benefit communication. Trends Food Sci Technol 30, 84–91.

[22] Neff RA , Chan IL & Smith KC (2009) Yesterday’s dinner, tomorrow’s weather, today’s news? US newspaper coverage of food system contributions to climate change. Public Health Nutr 12, 1006–1014. 18702838 10.1017/S1368980008003480

[23] Shoemaker PJ , Eichholz M , Kim E et al. (2001) Individual and routine forces in gatekeeping. J Mass Comm Q 78, 233–246.

[24] Shoemaker PJ , Vos TP & Reese SD (2009) Journalists as Gatekeepers. HandBook of Journalism Studies. New York: Routledge.

[25] Weitkamp E & Eidsvaag T (2014) Agenda building in media coverage of food research: superfoods coverage in UK national newspapers. J Pract 8, 871–886.

[26] Henderson J , Wilson AM , Webb T et al. (2017) The role of social media in communication about food risks. Br Food J 119, 453–467.

[27] Lee C-J , Nagler RH & Wang N (2018) Source-specific exposure to contradictory nutrition information: Documenting prevalence and effects on adverse cognitive and behavioral outcomes. Health Commun 33, 453–461. 28151010 10.1080/10410236.2016.1278495 PMC6102724

[28] Nagler RH (2014) Adverse outcomes associated with media exposure to contradictory nutrition messages. J Health Commun 19, 24–40. 24117281 10.1080/10810730.2013.798384 PMC4353569

[29] Arden MA (2010) Conflicting influences on UK mothers’ decisions to introduce solid foods to their infants. Maternal Child Nutr 6, 159–173. 10.1111/j.1740-8709.2009.00194.x PMC6860783 20624212

[30] Spiteri Cornish L & Moraes C (2015) The impact of consumer confusion on nutrition literacy and subsequent dietary behavior. Psychol Market 32, 558–574.

[31] British Nutrition Foundation (2018) Healthy Eating Week Survey. London: British Nutrition Foundation.

[32] BBC (2018) Food: Truth or Scare. https://www.bbc.co.uk/programmes/b08f17c0#:˜:text=Gloria%20Hunniford%20and%20Chris%20Bavin,headlines%20about%20Britain’s%20favourite%20foods (accessed January 2021).

[33] British Nutrition Foundation Facts behind the headlines. https://www.nutrition.org.uk/nutritioninthenews/headlines.html (accessed January 2021).

[34] NHS Behind the Headlines. https://www.nhs.uk/news/ (accessed November 2020).

[35] Gallotti R , Valle F , Castaldo N et al. (2020) Assessing the risks of ‘infodemics’ in response to COVID-19 epidemics. Nat Human Behav 4, 1285–1293. 33122812 10.1038/s41562-020-00994-6

[36] NHS Digital (2020) Statistics on Obesity, Physical Activity and Diet, England, 2020. https://digital.nhs.uk/data-and-information/publications/statistical/statistics-on-obesity-physical-activity-and-diet/england-2020 (accessed May 2020).

[37] Freisling H , Haas K & Elmadfa I (2013) Mass media nutrition information sources and associations with fruit and vegetable consumption among adolescents. Public Health Nutr 13, 269–275. 10.1017/S1368980009991297 19706216

[38] Zeeni N , Doumit R , Abi Kharma J et al. (2018) Media, technology use, and attitudes: associations with physical and mental well-being in youth with implications for evidence-based practice. Worldviews on evidence-based Nursing 15, 304–312. 10.1111/wvn.12298 29763998

[39] Tangcharoensathien V , Calleja N , Nguyen T et al. (2020) Framework for managing the COVID-19 infodemic: methods and results of an online, crowdsourced WHO technical consultation. J Med Internet Res 22, e19659. 32558655 10.2196/19659 PMC7332158

[40] Ha S & Lee YJ (2011) Determinants of consumer-driven healthcare. Int J Pharmaceut Healthcare Market 5, 8–24.

[41] Hesse BW , Nelson DE , Kreps GL et al. (2005) Trust and sources of health information: the impact of the Internet and its implications for health care providers: findings from the first Health Information National Trends Survey. Arch Intern Med 165, 2618–2624. 16344419 10.1001/archinte.165.22.2618

[42] Koch-Weser S , Bradshaw YS , Gualtieri L et al. (2010) The Internet as a health information source: findings from the 2007 Health Information National Trends Survey and implications for health communication. J Health Comm 15, 279–293. 10.1080/10810730.2010.522700 21154099

[43] Fletcher R , Kalogeropoulos A & Nielsen RK (2020) Trust in UK government and news media COVID-19 information down, concerns over misinformation from government and politicians up. Concerns Over Misinformation from Government and Politicians Up. https://reutersinstitute.politics.ox.ac.uk/trust-uk-government-and-news-media-covid-19-information-down-concerns-over-misinformation (accessed January 2021).

[44] Nagler RH , Vogel RI , Gollust SE et al. (2020) Public perceptions of conflicting information surrounding COVID-19: results from a nationally representative survey of US adults. PLoS One 15, e0240776. 33085719 10.1371/journal.pone.0240776 PMC7577476

[45] Barron E , Bakhai C , Kar P et al. (2020) Associations of type 1 and type 2 diabetes with COVID-19-related mortality in England: a whole-population study. Diabetes Endocrinol 8, 813–822. 10.1016/S2213-8587(20)30272-2 PMC7426088 32798472

[46] Hermida A , Fletcher F , Korell D et al. (2012) Share, like, recommend: decoding the social media news consumer. J Stud 13, 815–824.

[47] Picard RG & Yeo M (2011) Medical and Health News and Information in the UK Media: The Current State Of Knowledge. A Report of the Reuters Institute for the Study of Journalism. Wageningen: Wageningen Academic Publishers.

[48] Koelen M & van den Ban AW (2004) Health Education and Health Promotion. Wageningen: Wageningen Academic Publishers.

[49] Szwajcer EM , Hiddink GJ , Koelen M et al. (2005) Nutrition-related information-seeking behaviours before and throughout the course of pregnancy: consequences for nutrition communication. Eur J Clin Nutr 59, S57–S65. 16052197 10.1038/sj.ejcn.1602175

[50] Hendriks F , Kienhues D & Bromme R (2015) Measuring laypeople’s trust in experts in a digital age: the Muenster Epistemic Trustworthiness Inventory (METI). PLoS One 10, e0139309. 26474078 10.1371/journal.pone.0139309 PMC4608577

[51] McMullan M (2006) Patients using the Internet to obtain health information: how this affects the patient–health professional relationship. Patient Educ Counseling 63, 24–28. 10.1016/j.pec.2005.10.006 16406474

[52] Cooper BE , Lee WE , Goldacre BM et al. (2012) The quality of the evidence for dietary advice given in UK national newspapers. Public Underst Sci 21, 664–673. 23832153 10.1177/0963662511401782

[53] Carpenter DM , Elstad EA , Blalock SJ et al. (2014) Conflicting medication information: prevalence, sources, and relationship to medication adherence. J Health Comm 19, 67–81. 10.1080/10810730.2013.798380 PMC8989251 24015878

[54] Sillence E , Briggs P , Harris PR et al. (2007) How do patients evaluate and make use of online health information? Soc Sci Med 64, 1853–1862. 17328998 10.1016/j.socscimed.2007.01.012

[55] Menczer F & Hills T (2020) Information Overload Helps Fake News Spread, and Social Media Knows It: Scientific American. https://www.scientificamerican.com/article/information-overload-helps-fake-news-spread-and-social-media-knows-it/ (accessed December 2020).

