# Supplementary material for: Associations between conflicting nutrition information, nutrition confusion and backlash among consumers in the UK
Source: Public Health Nutr. 2021 Jan 12;24(5):914–23. doi: 10.1017/S1368980021000124 (PMC10195575; doi:10.1017/S1368980021000124)
Supplement: Supplementary file 1 [file S1368980021000124sup001.docx]

Supplementary Table 1: Factor loadings for nutrition backlash scale

|  | | **Factor 1** | | **Uniqueness** | |
| --- | --- | --- | --- | --- | --- |
| I am tired of hearing about what foods I should or should not eat. |  | 0.60 |  | 0.64 |  |
| Dietary recommendations are rarely useful. |  | 0.68 |  | 0.54 |  |
| Scientists really don’t know what foods are good for you. |  | 0.69 |  | 0.52 |  |
| Scientific research provides good guidance about the best foods to eat. (R) |  | . |  | 0.95 |  |
| I pay attention to new research on food and nutrition. (R) |  | . |  | 0.99 |  |
|  | | | | | |
| *Note.*  No rotation method applied. Items marked (R) have been reverse scored. Loadings less than .4 have been suppressed. | | | | | |

Supplementary Table 2: Exposure to conflicting nutritional information from multiple media channels in the past 12 months.

| Level of CNI Exposure | N | % |
| --- | --- | --- |
| Not at all | 118 | 17.5 |
| A little | 288 | 42.7 |
| Some | 179 | 26.5 |
| A lot | 90 | 13.3 |
